# Hypothermic oxygenated perfusion (HOPE) against cancer recurrence after liver transplantation for hepatocellular carcinoma—study protocol for an international multicenter randomized controlled trial (HOPE4Cancer)

**DOI:** 10.1186/s13063-025-09120-1

**Published:** 2025-09-26

**Authors:** Janina Eden, Philip C. Müller, Christoph Kuemmerli, Florian Peters, Tanja Litke, Anne Kranich, Andreas E. Kremer, Stefanie von Felten, Philipp Dutkowski, HOPE4Cancer Trial Investigators, Vincent E de Meijer, Lutz Fischer, Umberto Cillo, Riccardo De Carlis, Vincenzo Mazzaferro, Robert J Porte, Jacques Pirenne, Andrea Schlegel, James V Guarrera, Gabriela Berlakovich, Jiri Fronek, Georg Lurje, Jens Mittler, Moritz Drefs, Philippe Compagnon, Miriam Cortes-Cerisuelo, Joerg M Pollok, Barbara Fiore, Rebeca Sanabria-Mateos, Christoph Tschuor, Cornelius van Beekum, Ulf P Neumann, Gabriel C Oniscu, Povilas Ignatavicius, Xavier Muller, Diethard Monbaliu, Stefan Schneeberger, Piotr Domagala, Pål-Dag Line, Umberto Baccarani, Eliano Bonaccorsi, Rodrigo Figueiredo, Philipp Kron

**Affiliations:** 1 Department of Surgery, Section of HPB Surgery and Liver Transplantation, University of Groningen and University Medical Center Groningen, Groningen, the Netherlands; 2 Department of Surgery, Clarunis University Digestive Health Care Center, University Hospital Basel, Basel, Switzerland; 3 University of Basel, Basel, Switzerland; 4 Department of Clinical Research, University Hospital Basel, Basel, Switzerland; 5 OncoDrugConsult BV, Amsterdam, the Netherlands; 6 Department of Gastroenterology and Hepatology, University Hospital Zurich, Zurich, Switzerland; 7 Department of Biostatistics, Epidemiology, Biostatistics, and Prevention Institute, Faculty of Medicine, University of Zurich, Zurich, Switzerland

**Keywords:** Hepatocellular carcinoma, Perfusion, Liver transplantation, Organ preservation

## Abstract

**Background:**

A frequent and increasing indication for liver transplantation (LT) is hepatocellular carcinoma (HCC). However, despite strict selection criteria, HCC recurrence after LT occurs in a relevant proportion of patients and is associated with an unfavorable prognosis. Hypothermic oxygenated perfusion (HOPE) is a novel machine liver perfusion approach to optimize liver grafts before implantation and has been suggested to decrease graft inflammation with potential anti-cancer effects.

**Methods:**

HOPE4Cancer is an international, multicentric, parallel group, randomized controlled trial comparing HOPE performed after initial cold storage (intervention) with conventional cold storage alone (control) in a 1:1 allocation ratio. Adult recipients with proven HCC will be included for transplantation of a DBD (donation after brain death) Liver graft. The minimum perfusion duration is defined at 2 h and perfusion is generally continued until the recipient hepatectomy is completed. The conventional cold storage at 4 °C will be performed with a precooled preservation solution according to the local standard of care. The primary endpoint is defined as post-transplant HCC recurrence-free survival, i.e., the time interval a patient is alive without HCC recurrence after transplantation. Secondary endpoints are the single components of the events considered for the primary outcome (i.e., HCC recurrence, HCC-related death, death from any other causes than HCC), circulating tumor DNA, high-mobility-group-protein B1 in the blood, the Rejection Activity index, and the number of liver-related complications experienced by the patient.

**Discussion:**

HOPE4Cancer investigates if cold storage plus end-ischemically applied HOPE in DBD LT is superior to conventional cold storage of liver grafts in terms of post-transplant HCC recurrence-free survival. The results will indicate for the first time whether ex situ HOPE before transplant has an anti-cancer potential compared to transplantation of un-perfused livers.

**Trial registration:**

ClinicalTrials.gov NCT06717919. Registered on December 5, 2024.

## Administrative information

**Table:** Note: the numbers in curly brackets in this protocol refer to SPIRIT checklist item numbers. The order of the items has been modified to group similar items (see http://www.equator-network.org/reporting-guidelines/spirit-2013-statement-defining-standard-protocol-items-for-clinical-trials/).

Title {1}	Hypothermic oxygenated perfusion (HOPE) against cancer recurrence in HCC liver transplantation - International multicenter parallel group interventional RCT
Trial registration {2a and 2b}.	Clinicaltrials.gov NCT06717919. Registered on 5th December 2024.
Protocol version {3}	Version 1.1, 22.11.2024
Funding {4}	Swiss National Science Foundation (SNSF), grant number 33IC30_221622
Author details {5a}	Department of Surgery, Section of HPB Surgery and Liver Transplantation, University of Groningen and University Medical Center Groningen, Groningen, the Netherlands Department of Surgery, Clarunis University Digestive Health Care Center, University Hospital Basel, Basel, Switzerland Department of Clinical Research, University Basel, University Hospital Basel, Switzerland OncoDrucConsult BV, Amsterdam, the Netherlands Department of Gastroenterology and Hepatology, University Hospital Zurich, Zurich, Switzerland Department of Biostatistics, Epidemiology, Biostatistics, and Prevention Institute, University of Zurich, Hirschengraben 84, 8001 Zurich, Switzerland
Name and contact information for the trial sponsor {5b}	Prof. Philipp Dutkowski Department of Surgery, Clarunis University Digestive Health Care Centre Basel, University Hospital Basel Spitalstrasse 21 4031 Basel Switzerland e-mail:philipp.dutkowski@usb.c phone: 0041 61 777 7319
Role of sponsor {5c}	The funder and the sponsor institution had no role in the study design, collection, management, analysis, and interpretation of data, writing of the report and the decision to submit the report for publication.

## Introduction

### Background and rationale {6a}

**Fig. 1:**
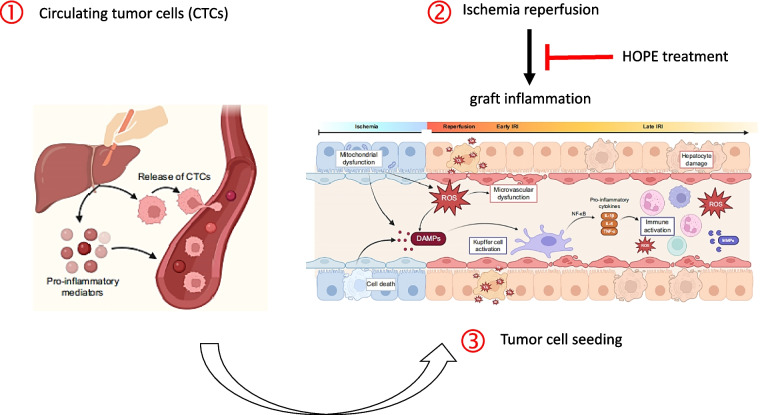
Postulated protective mechanism of machine liver perfusion against cancer recurrence. Modified from Maspero et al., JHPE 2023 [38]

Hepatocellular carcinoma (HCC) is the most common primary liver cancer, and liver transplantation for early HCC stages is the treatment of choice [1, 2]. Liver transplant results in excellent outcomes with overall 5-year survival rates of 70% [3]. However, HCC recurrence may occur after LT and is reported in up to 15% of cases, associated with poor outcome [4, 5]. The mechanisms for HCC recurrence after hepatectomy and liver transplantation (LT) are not yet fully understood. Several factors, including tumor seeding, unrecognized residual micro-lesions in lymph nodes, active hepatitis, increased liver regeneration, and immunosuppressive treatment, may promote cancer growth or recurrence [6]. In addition, ischemia reperfusion injury has been recognized as an important initial driver of microvascular dysfunction with subsequent tissue hypoxia and ongoing inflammation, which promotes tumor cell reseeding and growth [6]. Accordingly, in a cascade-like mechanism, ischemia reperfusion injury (IRI) leads to hepatic sinusoidal injury, which causes dysregulation of the hepatic microcirculatory barrier and activates cell signaling related to invasion and migration [7]. Hypoxia triggers gene upregulation and release of cytokines, involved in angiogenesis, cellular proliferation, growth, and adhesion. In the absence of oxygen, hypoxia-inducible transcription factor 1 (HIF-1a) binds to hypoxia-response elements, thereby upregulating the hypoxia-response gene expression, including vascular endothelial growth factor [8, 9]. Inflammatory mediators, including reactive oxygen species (ROS) and numerous cytokines (TNFα, IL-1, IL-6, IFN-y) released from various immune cells, induce epigenetic alterations in premalignant lesions and silence tumor suppressor genes [10, 11]. It is currently hypothesized that a key link between inflammation and cancer recurrence may originate from mitochondria. An early mitochondrial protection may therefore be decisive for decreasing tissue inflammation and subsequent tumor cell seeding (Fig. 1).

During the past 10 years, organ machine perfusion has regained clinical interest due to advantages compared to cold static storage. For example, machine liver perfusion reduces post-transplant complications and increases graft survival by improving early allograft function [12]. Hypothermic oxygenated perfusion (HOPE) has recently gained popularity due to its simplicity besides clinical efficacy. First, it is performed at the recipient center after organ transport, i.e., cold storage, and therefore does not change current practice in organ procurement. Secondly, it reduces significantly IRI by mitochondrial reprogramming [13–15]. Thirdly, it is particularly beneficial in extended criteria donor livers, such as steatotic livers and livers donated after circulatory death (DCD). Mechanistically, HOPE leads to full metabolization of accumulated succinate and NADH, together with replenished cellular ATP pools and improved function of mitochondrial complexes I–IV. This leads consecutively to decreased inflammation signals during implantation and may therefore potentially reduce also the risk of HCC recurrence [14, 16, 17]. Accordingly, a retrospective study reported a significant reduction of tumor recurrence rate from 25.7% in un-perfused donation after brain death (DBD) Livers compared to only 5.7% in HOPE-treated DCD organ recipients [14].

This study therefore aims at showing that post-transplant HCC recurrence-free survival (RFS) is improved by end-ischemically applied HOPE before transplantation, compared to standard conventional cold storage in patients who undergo liver transplantation for HCC.

### Objectives {7}

The overall objective of this trial is to investigate the anti-tumor effects of HOPE before liver transplantation in patients with HCC. The primary objective is to show that HCC recurrence-free survival is improved with HOPE compared to conventional cold storage alone in patients with liver transplantation for HCC.

### Trial design {8}

HOPE4Cancer is an international, multicentric, parallel group, superiority randomized controlled trial (RCT) comparing HOPE to conventional cold storage. Patients will be allocated in a 1:1 ratio.

## Methods: participants, interventions and outcomes

### Study setting {9}

**Fig. 2:**
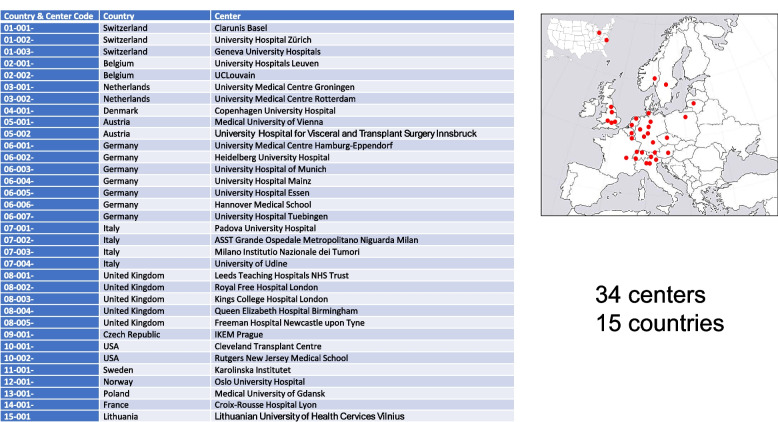
Study sites

The trial will be conducted in approximately 34 academic tertiary referral centers that have implemented HOPE before the start of the trial. Centers are in Switzerland, other European countries, and the USA (Fig. 2).

### Eligibility criteria {10}

The following are inclusion criteria for patients:

- Adult recipients (> 18 years old)
- Listed for liver transplantation
- Documented HCC (LIRADS-5 lesion in MRI or CT of the liver or biopsy proven) within up to seven criteria, i.e., HCC with seven as the sum of the diameter of the largest tumor (in cm) and the number of tumors at the timepoint of liver transplantation. This includes also patients beyond the up to seven criteria after successful downsizing of the HCC.
- Written informed consent for the trial

The following exclusion criteria will be applied:

- DCD liver grafts
- Combined liver transplants
- Partial liver transplants
- Combined or mixed hepatocellular cholangiocarcinoma or pure cholangiocarcinoma or other malignancies in histopathology of the liver explant
- Systemic antitumoral medical treatment with checkpoint inhibitors or multikinase inhibitors
- Post-transplant treatment with mTOR inhibitors
- Acute and unexpected medical contraindication
- Pregnancy
- Cold storage time of > 10 h (in both study arms)

### Who will take informed consent? {26a}

All participants will be provided a participant information sheet and a consent form describing the study and providing sufficient information for participants to make an informed decision about their participation in the study. The time frame given is at least 7 days. The formal consent of a participant, using the approved consent form, must be obtained before the participant is submitted to any study procedure. The investigators or a designee will take consent in the outpatient clinic.

### Additional consent provisions for collection and use of participant data and biological specimens {26b}

Participants will be asked in the consent form if they agree to the use of their data should they choose to withdraw from the trial. Participants will also be asked for permission for the research team to share relevant data with people from the universities and regulatory authorities taking part in the research. In addition, all participants will be asked whether they agree to the further use of health-related data or not.

## Interventions

### Explanation for the choice of comparators {6b}

Conventional cold storage after organ procurement has been the standard of care before the implementation of machine perfusion for liver grafts and is currently still the most commonly used method. It involves storage of the organ on ice. Every transplant center is familiar with the procedure and no additional training or material is required for the storage procedure in the control arm.

### Intervention description {11a}

**Fig. 3:**
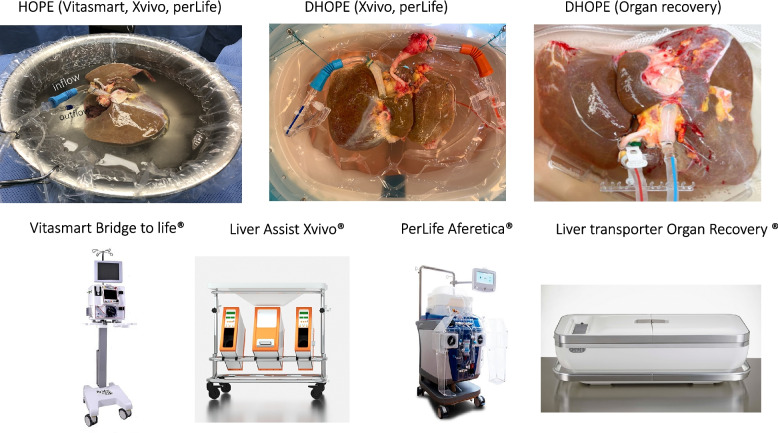
Machine perfusion technique and devices

All study centers will use either a VitaSmart®, Liver Assist®, or Perlife® device for machine liver perfusion (Fig. 3), with a pressure-controlled hypothermic oxygenated liver perfusion through the portal vein (HOPE) or through the portal vein and the hepatic artery (DHOPE). The rationale to choose VitaSmart® and Liver Assist® is based on their CE sign and the clinical experience during recent and ongoing RCTs [18–20] (NCT05045794). Perlife® also has a CE sign and is used currently in another RCT (NCT04644744). There are no RCTs comparing the effectivity of HOPE vs. DHOPE. However, recent cohort and experimental data show no differences between HOPE and DHOPE in terms of perfusion quality and also in terms of outcome [18, 21–23]. The oxygenation process of the perfusate is similar, as the same oxygenators are used. The perfusion flow in both procedures is pressure controlled. For the sake of simplicity, we therefore refer to HOPE/DHOPE as HOPE throughout the manuscript. The targeting pressure is 3–5 mmHg and the perfusate temperature is between 8 and 12 °C. The perfusate consists of 3L recirculating Belzer MPS® (Bridge to Life Ltd.) with active oxygenation (70–110 kPa). The minimum perfusion duration is defined as 2 h, while perfusion is generally continued until the recipient hepatectomy is completed, i.e., if the recipient hepatectomy takes longer, perfusion is extended. The absolute upper Limit for HOPE is 24 h according to recent publications [24, 25]. The perfusion device is routinely used in all participating centers. All machines used are certified for human use and controlled by transplant surgeons. Cooling of the perfusate is managed by a heat exchanger (Liver assist®) or ice (VitaSmart®); the perfusion flow is adjusted to 150–250 ml/min under pressure control (< 5 mm Hg). Commercially available, CE-certified and approved Belzer MPS solution will be used (Belzer MPS-UW-Machine perfusion solution, Bridge to Life®) as perfusate for machine perfusion (according to clinical routine).

### Criteria for discontinuing or modifying allocated interventions {11b}

If there are safety concerns or withdrawal of informed consent, patients are withdrawn from the study.

### Strategies to improve adherence to interventions {11c}

Minimal deviation from the protocol is expected due to the experience of the centers with both the intervention and the control procedures which are both part of the clinical routine in all centers. Nevertheless, cold storage time, the used preservation solution, the machine perfusion type, time, and oxygenation will be recorded and assessed to identify non-adherence.

### Relevant concomitant care permitted or prohibited during the trial {11d}

All concomitant care or interventions needed after transplantation are permitted, except that post-transplant mTOR inhibitors are prohibited.

### Provisions for post-trial care {30}

The post-transplant imaging for the HCC surveillance will be compensated. Otherwise, there is no additional study-related procedure and no deviation from the usual care after LT.

### Outcomes {12}

The primary outcome is post-transplant HCC recurrence-free survival, i.e., the time interval until either tumor recurrence is observed or the patient dies from any cause. HCC recurrence is assessed by computed tomography (CT) at 6, 12, 18, and 24 months (and every 6 months afterwards, if appropriate, until the end of the study), which will be checked for intrahepatic and extrahepatic masses according to the LiRADS classification. At least 24 months of follow-up duration for every patient is required. HCC recurrence is clinically highly relevant as only limited treatment options are available after liver transplantation. This time-to-event measurement will be aggregated as a hazard rate.

Secondary outcomes are:

- Single components of the events considered for the primary outcome, i.e., HCC recurrence (while alive), HCC-related death, and death from any other causes than HCC. As the primary outcome, each component will be analyzed as time to event (up to a minimum of 24 months) and aggregated as hazard rate.
- Circulating tumor DNA in blood (before transplantation, at discharge, and at 6 months after transplantation)
- High-mobility-group-protein-B1 (HMGB-1) in blood (at the time of transplantation and 1 week after transplantation)
- Rejection Activity index (RAI) in liver histology (biopsy) performed at 6 months post-transplantation
- Number of Liver-related complications experienced by the patient within 2 years

Other outcomes of interest are:

- Spatial transcriptomics of T cells (CD4 +, CD8 +) and monocytes (CD14 +) in liver biopsies at the end of implantation
- Laboratory parameters AST/ALT/bilirubin, AP, INR, measured daily during the first week after transplantation
- Early allograft dysfunction (EAD) based on laboratory parameters measured over week 1 after transplantation
- Parameters assessed in the liver explant of the patient (phenotyping of tumor infiltrating and tumor stroma immune cells in liver explants, RNA sequencing, metabolomics and spatial pathology in tumor and tumor stroma in liver explants)

### Participant timeline {13}

The participant timeline is shown in Table 1.

**Table 1:** Schedule of enrollment, intervention and assessments

Time point	Visit −1	Visit 0 OR	Visit 1 Day 1–6	Visit 2 Day 7	Visit 3 Discharge	Visit 4 6 months	Visit 5 12 months	Visit 6 18 months	Visit 7 24 months
Day/month		d0		d7		m6	m12	m18	m24
Visit window									
**Eligibility screens**									
**Demographic data**									
Gender	x								
Year of birth	x								
Body height	x								
Body weight	x								
Inclusion and exclusion criteria	x								
**Informed consent**	x								
**Allocation/randomization**		x							
**Intervention**									
Liver biopsy at the end of LT and after 6 months		x				x			
**Baseline variables**									
**Donor/recipient#**	x								
Transplant listing timepoint	x								
**Underlying liver disease** (Hep C, Hep B, Hep A, Hep E, NASH, ASH, PSC, PBC, autoimmune)	x								
**Donor information**									
BMI	x								
Donor risk index	x								
Cold ischemia time		x							
Preservation solution		x							
**Machine perfusion**									
Machine perfusion time		x							
Machine perfusion type		x							
Machine perfusion pressure		x							
Machine perfusion oxygenation		x							
Asservation of 10 ml machine perfusate		x							
**Blood values**									
ALT		x	x	x	x				x
AST		x	x	x	x				x
INR		x	x	x	x				x
Bilirubin		x	x	x	x				x
Alkaline phosphatase		x	x	x	x				x
HMBG-1 in blood*		x		x					
Circulating tumor DNA*		x			x	x			
MELD score		x							
Creatinine		x		x					x
Serum alpha-fetoprotein		x				x	x	x	x
**Intraoperative course**									
Number of RBC units during OLT		x							
Duration of OLT		x							
Type of OLT		x							
**Postoperative course**									
PNF				x					
EAD				x					
HAT				x	x	x	x	x	x
ICU stay					x				
Hospital stay					x				
Type of immunosuppressive therapy					x				
Tacrolimus level				x	x	x	x	x	x
Rejection Activity Index (RAI)						x			
**Tumor criteria in explanted liver**									
RNA sequencing				x					
Phenotyping				x					
Follow-up									
CT (abdominal and thoracic)						x	x	x	x
Number of liver-related complications			x	x	x	x	x	x	x
Clavien grading			x	x	x	x	x	x	x
Adverse events/safety	x	x	x	x	x	x	x	x	x

^#^Donor age, donor BMI, donor cause of death (CVI, trauma, hypoxia, cardiac arrest, other), recipient MELD score, INR, bilirubin, creatinine at transplant, balance of risk (BAR) score), AST, ALT before transplant

^*^Parameters with * have to be centrifugated within 4 h and stored at −80 °C until centralized analysis

### Sample size {14}

The sample size was calculated to show superiority of HOPE over conventional cold storage regarding HCC recurrence-free survival (time to HCC recurrence or death). The power was set to 80% at a significance level of 5%. Based on previous studies on Liver transplantation in patients with HCC, we expect that 15% of the patients in the control arm will develop tumor recurrence or die within 24 months [2, 26–28]. Based approximately on the results of a retrospective cohort study, we expect this risk to be reduced to 5% of the patients in the HOPE arm [14]. Under the assumption of exponential survival times, these rates correspond to a hazard ratio (HR) of 0.32. We expect an accrual period of 12 months and plan a minimum follow-up duration of 24 months (resulting in a total study duration of 36 months). Based on the method of Lachin and Foulkes and as implemented in the function *nSurvival* from the R package *gsDesign*, we will need 208 patients to be able to observe 26 events [29–31]. We expect a dropout rate of 5%, similar to the dropout rate of 4% observed in Schlegel et al. [18]. We will thus need to recruit 220 patients (110 per treatment arm).

### Recruitment {15}

Approximately 34 European and US centers will participate in this study (Fig. 1). All centers confirmed experience in machine Liver perfusion. The expected overall caseload in all centers is approximately 1400 Liver transplanted patients per year. Based on the expectation that one third of the transplanted population are HCC patients, i.e., 470 patients, and to recruit 50% of these cases, we estimate complete recruiting of 220 HCC Liver transplants within 1 year after the study start.

## Assignment of interventions: allocation

### Sequence generation {16a}

The computer-generated allocation sequence will be done in a 1:1 ratio using stratified block randomization with variable block sizes and stratification by study center. Stratification by center is important because there will be differences between centers with regard to the recipients of liver transplantation for HCC, due to different national or center-specific guidelines and criteria. Variable block sizes will better prevent surgeons from guessing the randomization sequence than fixed block sizes. Randomization lists will be created using the R package *blockrand* [32].

### Concealment mechanism {16b}

The centrally generated randomization lists (one per center) will be stored in the electronic data capture system SecuTrial® (interActive systems; iAs, Berlin). The lists will not be accessible by the study personnel who is screening and recruiting patients or entering data. The person who provided the randomization lists and the responsible data manager are the only persons with access to the randomization lists.

### Implementation {16c}

The R code to create the randomization lists will be prepared at the Department of Biostatistics of the Epidemiology, Biostatistics, and Prevention Institute, University of Zurich, Switzerland. An independent person at the Department of Clinical Research Basel, who is not otherwise involved in the trial, will then specify the random seed and run the code to produce the randomization lists. The random seed will determine the allocation and ensure reproducibility of the randomization lists. The randomization lists will then be handed over to the responsible data manager at the Department of Clinical Research, University Hospital Basel, Switzerland, who will store the lists in SecuTrial®. The allocation of a liver graft will be revealed to the transplant surgeon after the donor liver is accepted for transplantation to a particular recipient and is enrolled in the trial. More precisely, after inclusion and exclusion criteria are entered in SecuTrial®, the allocation will be done by clicking a “randomize” button in SecuTrial® by a designated member of the local study team.

## Assignment of interventions: blinding

### Who will be blinded {17a}

The allocation is blinded for all persons who perform the outcome assessments. Patients are also blinded. The statistician will be blinded for the sample size re-estimation during the interim analysis but will be unblinded for the final analysis. Surgeons are unblinded for the intervention as they are involved in the storage or perfusion process of the organs. However, the randomization result must not be listed on the surgery or any other report accessible to the study personnel not involved in the surgery to prevent unblinding.

### Procedure for unblinding if needed {17b}

If unblinding is required in the interest of a participant’s safety, the investigator will discuss the matter with the sponsor before unblinding. In a medical emergency, the principal investigator or delegate may unblind treating doctors via an “emergency unblinding” function in SecuTrial®, which requires password entry of the principal investigator or delegate and an additional confirmation for the unblinding. If the principal investigator is not available, the sponsor or a delegate from the project manager can be contacted at any time via phone for unblinding. When there is a breach of blinding, it will be recorded in the eCRF and the sponsor will be automatically notified. All unblinded cases will be included in the intention-to-treat analysis.

## Data collection and management

### Plans for assessment and collection of outcomes {18a}

Source data is sought from the following documents:

- Patient demographics and disease characteristics will be available in the clinic information systems.
- Donor information is routinely available to the transplant team during the organ allocation process from transplant coordinators and surgeons’ notes.
- Separate worksheets will be distributed for perfusion or cold storage details and (S)AE.
- Complications, blood results, and follow-up information will be collected from discharge notes and patient charts or nurse records as well as correspondence with other departments or family doctors. The blood samples for cDNA and HMGB-1 will be centrifuged within 2 h after sampling ad stored subsequently at −20 °C in the centers. The stored samples will be collected every 3 months and will be processed centrally at the University of Zurich.
- Histology and radiology reports will be collected for disease, graft information, and information about recurrence.

The sponsor is implementing and maintaining quality assurance and quality control systems with written SOPs and working instructions to ensure that trials are conducted, and data are generated, documented (record), and reported in compliance with the protocol, GCP, and applicable regulatory requirement(s).

A screening and enrollment log will be kept tracking the screening process and monitor the patient accrual and the progress of the study. Routine remote monitoring will regularly be conducted to check the completeness and plausibility of all data entered into the eCRF. Therefore, eCRFs will be kept current to reflect participant status at each phase during the study.

### Plans to promote participant retention and complete follow-up {18b}

Adherence to the intervention will be assured by training of the transplant surgeon if there is a deviation from the usual procedure at the respective center. After discharge, oncologic follow-up is according to recommended intervals and modalities and will be conducted by transplant surgeons, hepatologists, and oncologists.

### Data management {19}

The electronic data capture (EDC), GCP compliant software SecuTrial® will be used for data processing and management in the present project. SecuTrial® is provided and maintained by the Department of Clinical Research of the University Hospital Basel, Basel, Switzerland.

A current list with signatures and names of all authorized study personnel with access to the study data will be filed in the investigator site file and the trial master file, respectively. All the study files will be maintained by authorized study team members and are stored in a secured storage area. All data entry and correction activities as well as query resolution will be performed in each center by team members, who have the specific rights. SecuTrial® allows automatic data checks for completeness and plausibility. A built-in data logging tool (audit trail) ensures that any changes to the project or user activity (date and time stamp and user log) are continuously tracked in real time and accessible online or via downloadable audit table.

### Confidentiality {27}

SecuTrial® is an encrypted, online web tool and accessible only by secured log-in membership, to transfer the information from the source documents. It is assured that any authorized person, who may perform data entries and changes in the eCRF, can be identified.

In order to ensure patients’ confidentiality and compliance with data privacy regulations, a study-specific ID will be automatically generated for each patient, which allows pseudonymization of personal data. The List containing the Link between the patient pseudonym and the respective personal data will be stored appropriately by study coordinators and protected against unauthorized access. In case an audit or inspection is performed, all study data must be made available. The sponsor ensures that the study database will be securely stored until 20 years after the end of the study.

### Plans for collection, laboratory evaluation and storage of biological specimens for genetic or molecular analysis in this trial/future use {33}

All study data must be archived for a minimum of 20 years after study termination or premature termination of the clinical trial. All data will be stored on the server of the University Hospital Basel. The biological material (blood serum/plasma) will be stored at −80 °C for 5 years. Liver biopsies will be stored for 5 years.

## Statistical methods

### Statistical methods for analyzing primary and secondary outcomes {20a}

Detailed methodology for summaries and statistical analyses of the data collected in the trial will be documented in a separate statistical analysis plan. The statistical analysis plan will be finalized before database closure and will be under version control at the Department of Biostatistics, University of Zurich.

We will estimate the hazard ratio (HR) for the effect of HOPE versus conventional cold storage on the primary outcome and report the HR with a 95% confidence interval (CI) and a *p* value for the test of the null hypothesis of a HR of 1. To account for variability between centers, we will use a mixed-effects Cox model with treatment as a fixed factor and a random intercept per center, as implemented in the R package coxme, i.e., a frailty model with Gaussian frailty distribution [33]. The advantage of a mixed-effects Cox model over other approaches to account for between-center variability with time-to-event outcomes (e.g., center as fixed effect or stratification for center in standard Cox models) is that small numbers of patients and events per center are better handled [34]. It should be noted that the estimated HR will compare the hazard rates of two subjects, one treated with HOPE and one with conventional cold storage, randomly drawn from the same center (conditional HR, center-specific interpretation). We will assess the proportional hazards assumption by inspecting the scaled Schoenfeld residuals. HCC recurrence-free survival in the two trial arms will be graphically displayed by Kaplan–Meier curves. The analysis will be performed on the FAS (see item 20c). Patients who are lost to follow-up or withdraw from the trial will be right censored at their last follow-up visit.

As a sensitivity analysis for the primary outcome, we will estimate a HR that is further adjusted for prognostic factors for the primary outcome. The model described above will be fitted with additional baseline covariates, such as the score from the BAR (balance of risk) scoring system, vascular infiltration of the tumor, tumor grading, lymph node involvement, and downstaging treatment as additional covariates. However, we expect that the number of events will limit the number of variables that can be added to the model. We will therefore fit several covariate-adjusted models, each with a number of covariates that is appropriate. Further, to account for interval censoring of tumor recurrence (if observed at one visit, we only know it has occurred between this visit and the last visit), we will fit a mixed-effects Weibull survival model [35]. We will fit the model once ignoring the interval censoring (to assess the sensitivity of results to using this parametric model instead of a semiparametric Cox model) and once correctly accounting for interval censoring (to assess the sensitivity of results to accounting for interval censoring when using the parametric model).

Single components of the events considered for the primary outcome (tumor recurrence, HCC-related death, death from other causes) will be analyzed by cause-specific mixed-effects Cox models. We will account for competing risks by censoring patients with the competing event on the date of the competing event. We will use the Aalen-Johansen estimator to derive cumulative incidence function curves for all competing events. HMGB-1 on day 7 and circulating tumor DNA in blood at discharge and 6 months after transplantation will be analyzed by linear mixed-effects models, with corresponding measurements at baseline as covariates. The models will include a random intercept per center, and the one for circulating tumor DNA in blood will additionally contain a random intercept per patient, to account for the repeated outcome measurements. Log-transformation or a switch to a generalized linear mixed-effects model will be considered in case of violation of the normality assumption. Acute rejection index (ordinal outcome) will be compared between groups by a proportional odds model stratified by center [36]. The number of Liver-related complications experienced by the patient within 2 years will be analyzed the same way, since it is expected to be zero for a considerable proportion of the patients as in Schlegel et al. and thus analysis as a count is complicated [37]. As for the primary outcome, we will report effect sizes with 95% CI and *p* values for all secondary outcomes.

No statistical tests will be performed for other outcomes, but they will be descriptively compared between treatment arms.

### Interim analyses {21b}

When 165 patients are recruited (75% of 220 patients), a blinded sample size re-estimation will be performed. We will use the method proposed by Todd et al. for designs with flexible follow-up [38]. We will compare the average survival probability over both trial arms, S_new_(t), at t = 6, 12, 18, and 24 months of follow-up, from the data ad interim with the anticipated survival probability used for the original sample size estimation, S_orig_(t). The average difference (on the complementary log-log scale) will then be used to predict the overall tumor recurrence-free survival probability at 24 months, S_new_(24). We will re-estimate the required number of patients for the trial not only accounting for changes in survival probabilities but also for changes in the recruitment rate or in the dropout rate. The sample size will be increased if S_new_(t) > S_orig_(t) on average, i.e., if fewer events than expected are observed, given that the recruitment rate is as expected. A reduction of the sample size will not be considered. There are no statistical rules for stopping the trial earlier, neither for efficacy nor for futility.

### Methods for additional analyses (e.g., subgroup analyses) {20b}

Exploratory subgroup analyses are planned for the following baseline characteristics regarding the primary outcome: BAR score, vascular infiltration of the tumor, tumor grading, lymph node involvement, within/outside Milan criteria, tumor size, tumor nodule number, alpha-fetoprotein, downstaging treatment, and type of immunotherapy. For each subgroup variable, a mixed-effects Cox model will be fitted to the primary outcome. Treatment (HOPE vs. control), the subgroup variable, and the interaction between the subgroup variable and treatment will be included as fixed explanatory variables. A statistically significant interaction between one of the subgroup variables and treatment would indicate a different treatment effect in the corresponding subgroups or along the continuous baseline characteristic. For categorical subgroup variables, group-specific treatment effects (with 95% CI) will be computed, fitting a separate model for the corresponding subgroups, which will be reported together with the interaction *p* value. Since these subgroup analyses are exploratory, we do not plan to adjust *p* values for multiple testing.

To investigate associations of characteristics of the liver explant (e.g., tumor staging and grading, phenotype of tumor infiltrating and tumor stroma immune cells) and characteristics of the implant directly after transplantation (T cells and monocytes) with the primary outcome, descriptive tables will be created of these parameters in terms of the primary endpoint.

To assess the heterogeneity of the treatment effect among centers, Higgin’s *I*^2^ will be estimated [39]. To assess whether the effect of HOPE vs. conventional cold storage differs from that of DHOPE vs. conventional cold storage, the intervention group will be split into HOPE and dual HOPE, and the model described above for the primary outcome will be fitted with this three-level factor for treatment.

### Definition of analysis population relating to protocol non-adherence and any statistical methods to handle missing data {20c}

The full analysis set (FAS) will include all patients randomized to the trial who underwent liver transplantation. This means that patients in whom liver transplantation is not completed or who did not receive a full graft will be excluded, as these patients retrospectively violate inclusion criteria. Reasons for canceling a transplantation may be that the liver graft is not accepted for transplantation or that an unexpected disease is found in the recipient.

The FAS will then be analyzed in adherence to the intention-to-treat principle, analyzing patients according to their randomized treatment.

In terms of “intercurrent events,” we are using a “composite variable strategy” regarding the intercurrent event of death by integrating death in the primary outcome tumor recurrence-free survival and a “treatment policy strategy” regarding all patients who received transplantation [40].

For the primary outcome, we only expect missing data in patients who withdraw from the trial. Their primary outcome and secondary outcomes that are components of the primary outcome will be right censored at the time of withdrawal. The remaining secondary outcomes will be analyzed by complete case analysis. Due to the expected truncation of secondary outcomes by death, especially those outcomes measured later during follow-up, multiple imputation of missing values is not planned, since this would mean imputing data beyond death. Depending on the number of deaths and their balance between study arms, principal stratification may be used to additionally estimate the survivor average causal effect.

### Plans to give access to the full protocol, participant-level data and statistical code {31c}

The full protocol will be published alongside the results report as supplementary material. Metadata will be shared in a repository adhering to the FAIR principles [41]. Datasets and statistical codes will be made available upon reasonable request.

## Oversight and monitoring

### Composition of the coordinating center and trial steering committee {5d}

This trial is organized by the Department of Clinical Research, University Hospital Basel, in Basel in close collaboration with the Company OncoDrugConsult (ODC, Amsterdam), who manage organizational support and are responsible for monitoring and safety as well as for reporting.

### Composition of the data monitoring committee, its role and reporting structure {21a}

An Independent Data and Safety Monitoring Committee (DSMC) will be established to provide an independent review and assessment of the efficacy and safety data systematically and to safeguard the interest and safety of the participating subjects in the trial. The composition of the DSMC will consist of three independent individuals, including one oncologist and two surgeons. The DSMC is tasked with making a recommendation to the sponsor to continue or stop the trial, based on their assessment of safety information. Details are described in a DSMC charter.

### Adverse event reporting and harms {22}

Safety reporting in clinical investigations of medical devices shall be performed in line with the regulatory requirements. Events will be collected during all visits during and after LT. All adverse events will be classified into different categories (MDR Article 2(57)), i.e., adverse events (AE), adverse device effects (ADE), anticipated serious adverse effects (ASADE), device deficiencies (DD), incidents, new findings, serious adverse events (SAE), serious adverse device effects (SADE), serious incidents, and unanticipated serious adverse device effects (USADE).

### Frequency and plans for auditing trial conduct {23}

The investigator will allow the persons being responsible for the audit, which is independent from the sponsor and the investigators, or the inspection to have access to the source data/documents and to answer any questions arising. All parties involved will keep the patient data strictly confidential.

### Plans for communicating important protocol amendments to relevant parties (e.g., trial participants, ethical committees) {25}

Substantial amendments are only implemented after approval of the ethics committee.

Under emergency circumstances, deviations from the protocol to protect the rights, safety, and well-being of human participants may proceed without prior approval of the sponsor and the ethics committee. Such deviations will be reported to the clinical ethics committee as soon as possible. All non-substantial amendments are communicated to the ethics committee within the Annual Safety Report (ASR).

### Dissemination plans {31a}

This study will be published after closure of the database and complete assessment of all results (expected 6–12 months after closure of the database). The results of the study will be presented at national and international medical congresses on corresponding fields of interest and published in medical journals.

## Discussion

Machine perfusion for LT before implantation has gained significant interest within the last 5 years, due to its effect on graft optimization compared to conventional cold storage, and also due to the possibility to test and assess organ function before use.

Tumor recurrence after LT for HCC is a relevant clinical problem in around 15–20% of patients, and likely linked to the mobilization of circulating tumor cells (CTCs), activation of dormant micrometastases, or a combination of the two [42]. LT may cause major alterations in the local microenvironment and at the systemic level, with activation of pathways that favor the engraftment of residual tumor cells [6]. Accordingly, higher rates of tumor recurrence after LT have been reported with increasing cold storage or increasing warm ischemia times or also with increasing donor-related factors such as age over 60 years, a history of diabetes, body mass index > 35 kg/m^2^, and severe graft steatosis [43, 44]. Machine liver perfusion is therefore an interesting and novel option to counteract tumor seeding by decreasing initial graft inflammation. HOPE targets mitochondria and minimizes the release of danger signals, which decrease inflammasome activation and downstream injury with potential protection from tumor recurrence [14, 15]. Currently, only limited evidence from retrospective single-center studies is available on the effect of tumor recurrence by HOPE with inconsistent results. For example, tumor recurrence was lower in a HOPE cohort of DCD livers published by Mueller et al., while in a study by Rigo et al. the recurrence rate was unaffected [14, 17]. Based on these results, randomized, high-level evidence is urgently needed. As an ex situ graft treatment before implantation is a relatively easy and cheap procedure, an anti-cancer effect of machine perfusion, regardless of the perfusion mode, would have large clinical consequences in the field.

## Trial status

Protocol version: 1.1, 22.11.2024.

Recruitment start is August 2025. Recruitment completion is expected in 2026. Follow-up is planned to be completed in 2028.

## Data Availability

The final dataset will be available to the chief investigator, the trial coordinator and the statistician. After publication of the final findings report, the data will be made available upon reasonable request.

## Abbreviations

BAR: Balance of risk
DBD: Donation after brain death
DCD: Donation after circulatory death
DHOPE: Dual hypothermic oxygenated perfusion
DNA: Deoxyribonucleic acid
CI: Confidence interval
CT: Computer tomography
EAD: Early allograft dysfunction
eCRF: Electronic case report form
EDC: Electronic data capture
FAS: Full analysis set
GCP: Good Clinical Practice
HAT: Hepatic arterial thrombosis
HR: Hazard ratio
HCC: Hepatocellular carcinoma
HIF-1a: Hypoxia-inducible transcription factor 1
HMGB-1: High-mobility-group-protein B1
HOPE: Hypothermic oxygenated perfusion
IRI: Ischemia reperfusion injury
LIRADS: Liver Imaging Reporting and Data System
LT: Liver transplant
mTOR: Mechanistic target of rapamycin
OR: Operating room
PNF: Primary non-function
RAI: Rejection Activity Index
ROS: Reactive oxygen species
SOP: Standard operating procedure

## References

[1] Clavien PA, Lesurtel M, Bossuyt PM, Gores GJ, Langer B, Perrier A. Recommendations for liver transplantation for hepatocellular carcinoma: an international consensus conference report. Lancet Oncol. 2012;13:e11-22. 22047762 10.1016/S1470-2045(11)70175-9 PMC3417764

[2] Drefs M, Schoenberg MB, Börner N, Koliogiannis D, Koch DT, Schirren MJ, et al. Changes of long-term survival of resection and liver transplantation in hepatocellular carcinoma throughout the years: a meta-analysis. Eur J Surg Oncol. 2024;50:107952. 38237275 10.1016/j.ejso.2024.107952

[3] Magyar CTJ, Arteaga NF, Germani G, Karam VH, Adam R, Romagnoli R, et al. Recipient-donor sex constellation in liver transplantation for hepatocellular carcinoma-an ELTR study. Liver Int. 2025;45:e16178. 39564600 10.1111/liv.16178 PMC11669077

[4] Goldberg D, Mantero A, Newcomb C, Delgado C, Forde KA, Kaplan DE, et al. Predicting survival after liver transplantation in patients with hepatocellular carcinoma using the LiTES-HCC score. J Hepatol. 2021;74(6):1398–406. 33453328 10.1016/j.jhep.2020.12.021 PMC8137533

[5] Mazzaferro V, Llovet JM, Miceli R, Bhoori S, Schiavo M, Mariani L, et al. Predicting survival after liver transplantation in patients with hepatocellular carcinoma beyond the Milan criteria: a retrospective, exploratory analysis. Lancet Oncol. 2009;10:35–43. 19058754 10.1016/S1470-2045(08)70284-5

[6] Parente A, Flores Carvalho M, Eden J, Dutkowski P, Schlegel A. Mitochondria and cancer recurrence after liver transplantation-what is the benefit of machine perfusion? Int J Mol Sci. 2022. 10.3390/ijms23179747. 36077144 10.3390/ijms23179747 PMC9456431

[7] Li CX, Man K, Lo CM. The impact of liver graft injury on cancer recurrence posttransplantation. Transplantation. 2017;101:2665–70. 28665890 10.1097/TP.0000000000001844 PMC6319559

[8] Luo D, Wang Z, Wu J, Jiang C, Wu J. The role of hypoxia inducible factor-1 in hepatocellular carcinoma. Biomed Res Int. 2014;2014:409272. 25101278 10.1155/2014/409272 PMC4101982

[9] Jiao M, Nan KJ. Activation of PI3 kinase/Akt/HIF-1α pathway contributes to hypoxia-induced epithelial-mesenchymal transition and chemoresistance in hepatocellular carcinoma. Int J Oncol. 2012;40:461–8. 21922131 10.3892/ijo.2011.1197

[10] He G, Karin M. NF-κB and STAT3 - key players in liver inflammation and cancer. Cell Res. 2011;21:159–68. 21187858 10.1038/cr.2010.183 PMC3193410

[11] Ghouri YA, Mian I, Rowe JH. Review of hepatocellular carcinoma: epidemiology, etiology, and carcinogenesis. J Carcinog. 2017;16:1. 28694740 10.4103/jcar.JCar_9_16 PMC5490340

[12] Parente A, Tirotta F, Pini A, Eden J, Dondossola D, Manzia TM, et al. Machine perfusion techniques for liver transplantation - a meta-analysis of the first seven randomized-controlled trials. J Hepatol. 2023;79:1201–13. 37302578 10.1016/j.jhep.2023.05.027

[13] Schlegel A, Porte RJ, Dutkowski P. Protective mechanisms and current clinical evidence of hypothermic oxygenated machine perfusion (HOPE) in preventing post-transplant cholangiopathy. J Hepatol. 2022;76:1330–47. 35589254 10.1016/j.jhep.2022.01.024

[14] Mueller M, Kalisvaart M, O’Rourke J, Shetty S, Parente A, Muller X, et al. Hypothermic oxygenated liver perfusion (HOPE) prevents tumor recurrence in liver transplantation from donation after circulatory death. Ann Surg. 2020;272:759–65. 32889870 10.1097/SLA.0000000000004258

[15] Kron P, Schlegel A, Mancina L, Clavien PA, Dutkowski P. Hypothermic oxygenated perfusion (HOPE) for fatty liver grafts in rats and humans. J Hepatol 2017. 10.1016/j.jhep.2017.08.028 28870676

[16] Dajti G, Germinario G, Prosperi E, Siniscalchi A, Vasuri F, Valente S, et al. The role of cold ischemia time and hypothermic perfusion in predicting early hepatocellular carcinoma recurrences after liver transplantation. Artif Organs. 2024;48:619–25. 38270476 10.1111/aor.14715

[17] Rigo F, De Stefano N, Patrono D, De Donato V, Campi L, Turturica D, et al. Impact of hypothermic oxygenated machine perfusion on hepatocellular carcinoma recurrence after liver transplantation. J Pers Med. 2023. 10.3390/jpm13050703. 37240873 10.3390/jpm13050703 PMC10220870

[18] Schlegel A, Mueller M, Muller X, Eden J, Panconesi R, von Felten S, et al. A multicenter randomized-controlled trial of hypothermic oxygenated perfusion (HOPE) for human liver grafts before transplantation. J Hepatol. 2023;78:783–93. 36681160 10.1016/j.jhep.2022.12.030

[19] van Rijn R, Schurink IJ, de Vries Y, van den Berg AP, Cortes Cerisuelo M, Darwish Murad S, et al. Hypothermic machine perfusion in liver transplantation - a randomized trial. N Engl J Med. 2021;384:1391–401. 33626248 10.1056/NEJMoa2031532

[20] Ravaioli M, Germinario G, Dajti G, Sessa M, Vasuri F, Siniscalchi A, et al. Hypothermic oxygenated perfusion in extended criteria donor liver transplantation-a randomized clinical trial. Am J Transplant. 2022;22:2401–8. 35671067 10.1111/ajt.17115 PMC9796786

[21] de Vries Y, Brüggenwirth IMA, Karangwa SA, von Meijenfeldt FA, van Leeuwen OB, Burlage LC, et al. Dual versus single oxygenated hypothermic machine perfusion of porcine livers: impact on hepatobiliary and endothelial cell injury. Transplant Direct. 2021;7:e741. 34386578 10.1097/TXD.0000000000001184 PMC8354629

[22] Koch DT, Tamai M, Schirren M, Drefs M, Jacobi S, Lange CM, et al. Mono-HOPE versus dual-HOPE in liver transplantation: a propensity score-matched evaluation of early graft outcome. Transpl Int. 2025;38:13891. 39974599 10.3389/ti.2025.13891 PMC11835512

[23] Eden J, Brüggenwirth IMA, Berlakovich G, Buchholz BM, Botea F, Camagni S, et al. Long-term outcomes after hypothermic oxygenated machine perfusion and transplantation of 1,202 donor livers in a real-world setting (HOPE-REAL study). J Hepatol. 2025;82:97–106. 38969242 10.1016/j.jhep.2024.06.035

[24] Brüggenwirth IMA, Lantinga VA, Lascaris B, Thorne AM, Meerdink M, de Kleine RH, et al. Prolonged hypothermic machine perfusion enables daytime liver transplantation - an IDEAL stage 2 prospective clinical trial. eClinicalMedicine. 2024;68:102411. 38235423 10.1016/j.eclinm.2023.102411 PMC10789636

[25] Brüggenwirth IMA, Mueller M, Lantinga VA, Camagni S, De Carlis R, De Carlis L, et al. Prolonged preservation by hypothermic machine perfusion facilitates logistics in liver transplantation: a European observational cohort study. Am J Transplant. 2022;22:1842–51. 35315202 10.1111/ajt.17037 PMC9540892

[26] Mazzaferro V, Sposito C, Zhou J, Pinna AD, De Carlis L, Fan J, et al. Metroticket 2.0 model for analysis of competing risks of death after liver transplantation for hepatocellular carcinoma. Gastroenterology. 2018;154:128–39. 28989060 10.1053/j.gastro.2017.09.025

[27] Kardashian A, Florman SS, Haydel B, Ruiz RM, Klintmalm GB, Lee DD, et al. Liver transplantation outcomes in a U.S. multicenter cohort of 789 patients with hepatocellular carcinoma presenting beyond Milan criteria. Hepatology. 2020;72:2014–28. 32124453 10.1002/hep.31210

[28] Wehrle CJ, Raj R, Maspero M, Satish S, Eghtesad B, Pita A, et al. Risk assessment in liver transplantation for hepatocellular carcinoma: long-term follow-up of a two-centre experience. Int J Surg. 2024;110:2818–31. 38241354 10.1097/JS9.0000000000001104 PMC11093438

[29] Lachin JM, Foulkes MA. Evaluation of sample size and power for analyses of survival with allowance for nonuniform patient entry, losses to follow-up, noncompliance, and stratification. Biometrics. 1986;42:507–19. 3567285

[30] Anderson KM, Guo Z, Zhao J, Sun LZ. A unified framework for weighted parametric group sequential design. Biom J. 2022;64:1219–39. 35704510 10.1002/bimj.202100085

[31] R Core Team (2023). R: a language and environment for statistical computing. R Foundation for Statistical Computing, Vienna, Austria. URL https://www.R-project.org/.

[32] Snow G, Snow M. Package “blockrand.” The comprehensive R archive network. 2013. In; 2020.

[33] Therneau TM (2022). coxme: mixed effects cox models. R package version 2.2-18.1. URL https://CRAN.R-project.org/package=coxme

[34] Munda M, Legrand C. Adjusting for centre heterogeneity in multicentre clinical trials with a time-to-event outcome. Pharm Stat. 2014;13:145–52. 24523155 10.1002/pst.1612

[35] Tamási B, Hothorn T. tramME: mixed-effects transformation models using Template Model Builder. R J. 2021. 10.32614/RJ-2021-075.

[36] Buri M, Curt A, Steeves J, Hothorn T. Baseline-adjusted proportional odds models for the quantification of treatment effects in trials with ordinal sum score outcomes. BMC Med Res Methodol. 2020;20:104. 32375705 10.1186/s12874-020-00984-2 PMC7204322

[37] Todd S, Valdés-Márquez E, West J. A practical comparison of blinded methods for sample size reviews in survival data clinical trials. Pharm Stat. 2012;11:141–8. 22337635 10.1002/pst.516

[38] Hemming K, Hughes JP, McKenzie JE, Forbes AB. Extending the I-squared statistic to describe treatment effect heterogeneity in cluster, multi-centre randomized trials and individual patient data meta-analysis. Stat Methods Med Res. 2021;30:376–95. 32955403 10.1177/0962280220948550 PMC8173367

[39] ICH E9 (R1) (2020). Addendum on estimands and sensitivity analysis in clinical trials to the guideline on statistical principles for clinical trials. Tech. Rep. Step 5 (17 February 2020). https://www.ema.europa.eu/en/documents/scientific-guideline/ich-e9-r1-addendum-estimands-sensitivity-analysis-clinical-trials-guideline-statistical-principles_en.pdf.

[40] Wilkinson MD, Dumontier M, Aalbersberg IJ, Appleton G, Axton M, Baak A, et al. The FAIR guiding principles for scientific data management and stewardship. Sci Data. 2016;3:160018. 26978244 10.1038/sdata.2016.18 PMC4792175

[41] Maspero M, Yilmaz S, Cazzaniga B, Raj R, Ali K, Mazzaferro V, et al. The role of ischaemia-reperfusion injury and liver regeneration in hepatic tumour recurrence. JHEP Rep. 2023;5:100846. 37771368 10.1016/j.jhepr.2023.100846 PMC10523008

[42] Grąt M, Krawczyk M, Wronka KM, Stypułkowski J, Lewandowski Z, Wasilewicz M, et al. Ischemia-reperfusion injury and the risk of hepatocellular carcinoma recurrence after deceased donor liver transplantation. Sci Rep. 2018;8:8935. 29895820 10.1038/s41598-018-27319-y PMC5997656

[43] Nagai S, Yoshida A, Facciuto M, Moonka D, Abouljoud MS, Schwartz ME, et al. Ischemia time impacts recurrence of hepatocellular carcinoma after liver transplantation. Hepatology. 2015;61:895–904. 25099130 10.1002/hep.27358

[44] Kornberg A, Witt U, Kornberg J, Friess H, Thrum K. Extended ischemia times promote risk of HCC recurrence in liver transplant patients. Dig Dis Sci. 2015;60:2832–9. 25630421 10.1007/s10620-015-3541-z

